# Distinct monkeypox virus lineages co-circulating in humans before 2022

**DOI:** 10.1038/s41591-023-02456-8

**Published:** 2023-09-14

**Authors:** Nnaemeka Ndodo, Jonathan Ashcroft, Kuiama Lewandowski, Adesola Yinka-Ogunleye, Chimaobi Chukwu, Adama Ahmad, David King, Afolabi Akinpelu, Carlos Maluquer de Motes, Paolo Ribeca, Rebecca P. Sumner, Andrew Rambaut, Michael Chester, Tom Maishman, Oluwafemi Bamidele, Nwando Mba, Olajumoke Babatunde, Olusola Aruna, Steven T. Pullan, Benedict Gannon, Colin S. Brown, Chikwe Ihekweazu, Ifedayo Adetifa, David O. Ulaeto

**Affiliations:** 1 Nigeria Centre for Disease Control, Abuja, Nigeria; 2 UK Public Health Rapid Support Team, UK Health Security Agency/London School of Hygiene & Tropical Medicine, London, UK; 3 UK Health Security Agency, Research & Evaluation Services, Porton Down, UK; 4 CBR Division, Defence Science and Technology Laboratory, Salisbury, UK; 5 Department of Microbial Sciences, School of Biosciences and Medicine, University of Surrey, Guildford, UK; 6 UK Health Security Agency, London, UK; 7 Biomathematics and Statistics Scotland, Edinburgh, UK; 8 Institute of Evolutionary Biology, University of Edinburgh, Edinburgh, UK; 9 UK Health Security Agency, International Health Regulations (IHR) Strengthening Project, British High Commission, Abuja, Nigeria

**Keywords:** Pox virus, Viral infection

## Abstract

The 2022 global mpox outbreak raises questions about how this zoonotic disease established effective human-to-human transmission and its potential for further adaptation. The 2022 outbreak virus is related to an ongoing outbreak in Nigeria originally reported in 2017, but the evolutionary path linking the two remains unclear due to a lack of genomic data between 2018, when virus exportations from Nigeria were first recorded, and 2022, when the global mpox outbreak began. Here, 18 viral genomes obtained from patients across southern Nigeria in 2019–2020 reveal multiple lineages of monkeypox virus (MPXV) co-circulated in humans for several years before 2022, with progressive accumulation of mutations consistent with APOBEC3 activity over time. We identify Nigerian A.2 lineage isolates, confirming the lineage that has been multiply exported to North America independently of the 2022 outbreak originated in Nigeria, and that it has persisted by human-to-human transmission in Nigeria for more than 2 years before its latest exportation. Finally, we identify a lineage-defining APOBEC3-style mutation in all A.2 isolates that disrupts gene *A46R*, encoding a viral innate immune modulator. Collectively, our data demonstrate MPXV capacity for sustained diversification within humans, including mutations that may be consistent with established mechanisms of poxvirus adaptation.

## Abstract (web summary)

Analysis of monkeypox virus (MPXV) isolates circulating in Nigeria before the 2022 global outbreak of MPXV sheds light on the diversification of the virus that eventually gave rise to the 2022 MPXV lineage.

## Main

MPXV is an orthopoxvirus (OPXV) endemic to West and Central Africa, circulating in one or more rodent species with epizootic infections of monkeys and chimpanzees, and zoonotic infections of humans. Human monkeypox, now known as mpox^1^, is traditionally a rash illness similar to smallpox with inefficient human-to-human transmission^2^. In addition, mpox is thought to have been suppressed by smallpox vaccination^2^. With smallpox eradication and the cessation of vaccination across large parts of Africa in 1980, the human niche has opened, and as anti-smallpox immunity has waned, the incidence of mpox zoonosis has steadily increased^3^. Most of these infections occurred in Central Africa by viruses genetically grouped as clade I, whereas a few occurred in West Africa by clade II viruses. In 2017, Nigeria reported its first mpox outbreak in nearly 40 years. The outbreak is ongoing, with tens of confirmed cases per year^4–6^. As a result, a few cases of mpox were detected between 2018 and 2021 in individuals traveling from Nigeria to the United Kingdom, Israel, Singapore and the United States. None of these early cases in non-endemic countries resulted in onwards transmission, except for one case in the United Kingdom resulting in transmission in a hospital setting^6–10^. In 2022, an ongoing global outbreak occurred in individuals with no history of travel to Africa, with extended human-to-human transmission and extensive virus genome sequencing^11^. Although virus genome sequences from the earlier exportations are available, there are very few sequences from human cases in Nigeria from 2017 to 2022. Such sequences are critical to bridge the gap in our understanding of how the outbreak developed from an initial zoonosis in or shortly before 2017 before the emergence of the current global strain, and the origins of viruses that continue to circulate and be exported from Nigeria independent of the global outbreak.

The global outbreak is concentrated among men-who-have-sex-with-men (MSM) communities^9,11^, establishing effective transmission chains and suggesting a newly predominant route of transmission between humans without reintroduction from animal reservoirs^12^. Initial analysis of global outbreak genome sequences revealed these formed a separate B.1 lineage within clade IIb, descending from the 2017 Nigerian outbreak lineage (lineage A) and proposed to have a single origin^13^. In addition, a third lineage (lineage A.2) has recently been described from three cases introduced to North America between 2021 and 2022, independently of the global outbreak^14^. As a zoonotic virus successfully adopting non-zoonotic transmission between humans, MPXV is unlikely to be optimally adapted to survival and transmission across its new host. Instead, adaptive mutations are to be expected along with dispersal and divergence driven by founder effects and selection pressure^12^.

How lineage A has evolved from its initial zoonotic jump eventually resulting in the genesis of lineages A.2 and B.1 remains obscure. Here, we have analyzed 18 MPXV genomes from individuals in Nigeria in 2019 and 2020, and possibly the only clade II mpox genomes available from this critical period in the dispersion and diversification of mpox in humans. Although lineage B.1 has more mutations than lineage A relative to the presumed ancestor, the data reveal there is more variability within lineage A than within B.1, with all B.1 isolates being closely related to each other^13^, and identify at least three independent clusters in lineage A, all with mutations compatible with the action of gene-modifying enzyme APOBEC3. We show lineage A.2 viruses circulating in humans more than a year before the first exportation of A.2 to North America, and describe a lineage A.2 defining APOBEC3-style mutation in a known OPXV immune modulator gene. Strikingly, this mutation is consistent with described mechanisms of OPXV adaption to new hosts. Our findings demonstrate that as of 2019, multiple variants of MPXV were circulating in humans in West Africa with a pattern of APOBEC3-like mutations that we suggest are consistent with diversification and sustained non-zoonotic transmission.

## Results

### Heterogeneity of monkeypox virus isolates before 2022

**Fig. 1:**
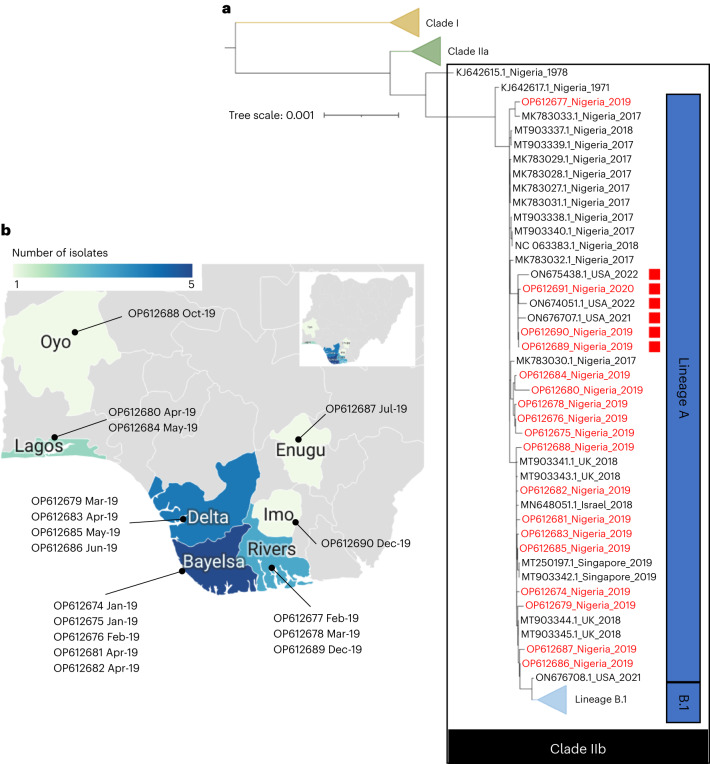
Phylogenetic analysis and geographic distribution MPXV genomes. **a**, Phylogenetic tree of clade II MPXV genomes. Genomes described in this study are highlighted in red and those with a disrupted *A46R* gene (lineage A.2) are marked with a red square. Published lineage B.1 genomes from the current international outbreak are depicted in a single cluster in gray. The cutoff date for inclusion of newly published genomes was 18 June 2022. **b**, Expanded map created with Datawrapper software (https://www.datawrapper.de/) showing the distribution of cases from which the genomes described in the study were isolated, across southern states of Nigeria. Geographic metadata were not available for OP612691.

An initial phylogenetic tree demonstrates the 18 sequences are from clade IIb (refs. ^15,16^) and cluster within lineage A (Fig. 1a). The sequences were from samples taken between January 2019 and January 2020, widely distributed across the southern part of Nigeria (Fig. 1b). We used the 1971 KJ642617 sequence^17,18^ as a reference, because it is the phylogenetically closest primary zoonotic isolate to lineage A, and the initial zoonotic progenitor of lineage A was never identified. A total of 187 polymorphisms, 149 of which are single-nucleotide polymorphisms (SNPs), were identified relative to KJ642617 (Supplementary Table 1). Of these, 35 are common to 17 of the genomes. This finding suggests mutation and potentially adaption in infected people, assuming all human cases since 2017 stem from a single zoonotic crossover. The identified polymorphisms are distributed throughout the genome and 84 are associated with alterations to protein primary sequence (Supplementary Table 1). They include both uncharacterized genes and well-characterized genes/proteins, including the virus F13 membrane protein (two instances of synonymous SNP) and the E9 DNA polymerase (two instances of non-synonymous missense SNP), which are the targets of the licensed smallpox antivirals tecovirimat and brincidofovir, respectively.

The distribution of mutations across the 18 genomes suggests that MPXV circulating in humans in Nigeria is diverging into subpopulations, which may be partially isolated from each other through demographics and dynamics of transmission. Available sequences from lineage B.1 form a distinct branch from this cluster (Fig. 1a), with a much lower baseline variation than the wider clade II population that has been causing human infections in Nigeria since at least 2017. Altogether, our analysis demonstrates the existence of divergent variants co-circulating widely in Nigeria before the emergence of lineage B.1.

### Mutations in putative APOBEC3 motifs

**Fig. 2:**
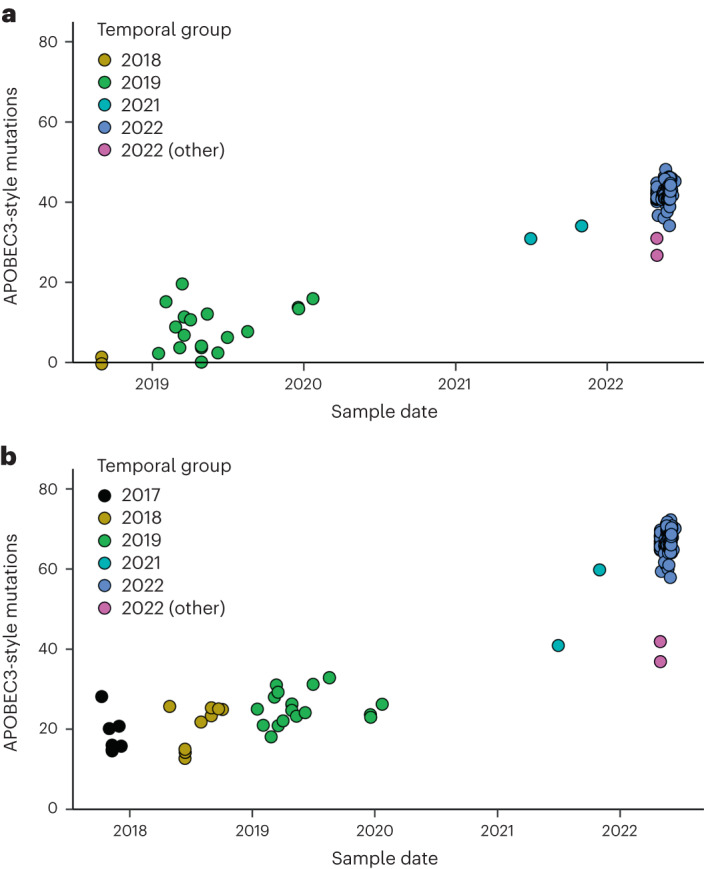
Frequency of APOBEC3-style mutations in clade II genomes. **a**,**b**, APOBEC3-style mutation frequency in clade II genomes isolated since 2019, using the UK 2018 MT903345 isolate as a baseline (**a**), and in clade II genomes isolated since 2017 using the Nigeria 1971 KJ642617 as a baseline (**b**). Genomes are placed in temporal groups by year of isolation except for OP612691, which is included in the 2019 group but was isolated in January 2020. ‘Other’ refers to lineage A.2 genomes isolated in 2022 in the United States, which are phylogenetically separated from the majority of sequences in the international outbreak and arise from a separate lineage within clade II.

**Fig. 3:**
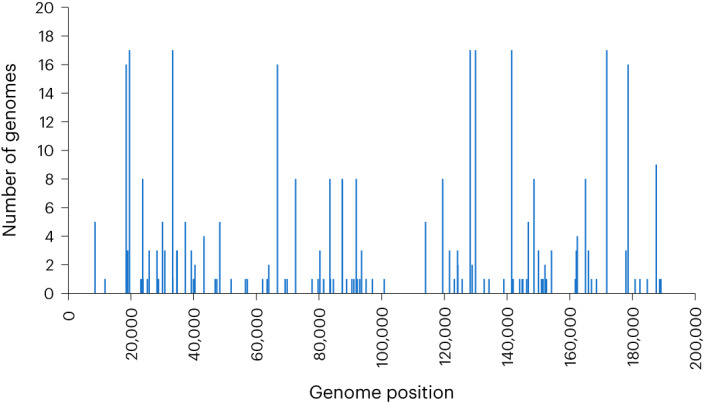
Distribution of APOBEC3-style mutations across the genome. The linear position of all APOBEC3-style mutations for all 18 isolates described in this study is plotted, with the number of isolates with a given mutation shown on the vertical axis. Genome length is 195 kb.

Analysis of multiple genomes from lineage B.1 has identified 47 shared single-nucleotide mutations relative to the 2018 UK reference genome MT903345, which represents an early lineage A exportation from Nigeria^13,19^. Of these, 45 were compatible with the action of APOBEC3 cytidine deaminase activity^19^, and it has been suggested this could provide the basis for a molecular clock to calculate the origin of the initial zoonotic crossover^20^. APOBEC3-style mutations described so far in the B.1 lineage occur at TC dinucleotides, with the C nucleotide deaminated to T^13,19^. Using the same MT903345 genome for a baseline, all 18 of the sequences described here have APOBEC3-style mutations, but at a lower frequency (median 8.5) than in the 2022 sequences from lineage B.1 (median 42; Fig. 2a). This difference was statistically significant (*P* = 8.37 × 10^−14^, Kruskal–Wallis test; Supplementary Table 2). There is considerable variation among the 18 genomes described here with respect to APOBEC3-style mutations, with the mutations occurring at different places in different genomes (Fig. 3 and Supplementary Table 1). Although none of the viruses in this study are directly ancestral to the B.1 lineage, OP612686 and OP612687 share the most APOBEC3-style mutations with genomes from B.1 and are also closest to B.1 sequences on the phylogenetic tree. This indicates that sustained non-zoonotic transmission may be facilitating MPXV acquiring APOBEC3-style mutations. The relatively low frequency of APOBEC3-style mutations observed in our sequences relative to those described for lineage B.1 could be a function of random accumulation over time, or alternatively indicate that APOBEC3 activity enabled rapid adaptation during the international outbreak. We therefore repeated this analysis as a comparison of the 18 genomes, and MT903345, against the 1971 KJ642617 genome, an isolated case without onward human transmission that likely represents a single zoonotic jump^17^. This analysis demonstrated a higher frequency of APOBEC3-style mutations in the 18 genomes (median 25), and a significant frequency in MT903345 and other genomes isolated in 2018 (median 22.5) (refs. ^6,21^). The median frequency of APOBEC3-style mutations in lineage B.1 genomes rose to 67 when compared with KJ642617 (Fig. 2b and Supplementary Table 3). Although there is a year-on-year increase in APOBEC3-style mutations from 2017 onwards, the only statistically significant differences are between 2022 and the other years. Pairwise comparisons between years 2017 to 2021 were not significant (Supplementary Table 4). This raises the possibility that the higher frequency of APOBEC3-style mutations in the B.1 2022 temporal group might involve more than random accumulation through continuous non-zoonotic transmission and may not be indicative of the number of sequential transmissions in the chain from the initial zoonotic parent virus. However, it should be noted that these comparisons do not account for any dependencies of the data such as shared ancestry, and so the statistical analysis should be interpreted with caution. It should also be noted that the 2022 outbreak appears to involve transmission in a more restricted but highly mobile demographic, predominantly via primary rash or skin lesions. This is expected to accelerate transmission chains^12^, which would affect the apparent rate of accumulation of APOBEC3-style mutations. This bears further analysis against genomes from Nigeria concurrent with the expansion of lineage B.1 and, if available, genomes from human isolates taken in 2020 and 2021. In this context, it is interesting that two genomes isolated in the United States in 2022 (ON674051 and ON675438; ‘2022 other’ in Fig. 2 and Supplementary Tables 2 and 3) from importations unrelated to the wider global outbreak and now designated lineage A.2 (ref. ^14^), have a lower frequency of APOBEC3-style mutations than B.1 isolates.

### Lineage A.2 in West Africa: an additional circulating variant of MPXV

A recent study of MPXV genomes from individuals in the United States before and concurrent with the global outbreak but directly imported from West Africa, has demonstrated that multiple clade II viruses have been found outside Africa since 2017, independent of the global B.1 lineage^14^. In particular, three clade IIb viruses from the United States cluster closely in a phylogenetic tree and are now designated as lineage A.2. The history of these three importations indicates they were independently exported from Nigeria over a period of 10 months from July 2021 to May 2022, suggesting this lineage was circulating efficiently in Nigeria before the global B.1 outbreak, and it is of note that one of the importations in May 2022 was a female who recently returned from Nigeria^14^.

Three of the genomes in this study cluster on the same branch of the phylogenetic tree as the United States A.2 genomes and are thus part of this lineage (Fig. 1a). Importantly, these three Nigerian genomes were isolated between December 2019 and January 2020. This strongly suggests that lineage A.2 originated in West Africa and extends the period for which the lineage is known to have circulated in humans to 30 months, during which it has achieved multiple exportations to North America.

Analysis of the six genomes now available for lineage A.2 shows 59 SNPs that are unique to any one of the six in comparison with the others, of which 54 (91.5%) are consistent with APOBEC3 action. Five of the six possess at least one SNP not found in any of the others, with OP612690 (67 SNPs relative to KJ642617) having only SNPs that are common to other members. OP612689 and OP612690 have one and two unique SNPs, respectively, while the later three genomes from July 2021 and May 2022 in the United States have 21, 19 and 19 SNPs that are unique to them relative to the other genomes in the lineage. Although there are only six genomes for comparison, the distribution of mutations between them indicate that two of the three lineage A.2 genomes presented in this study are not directly ancestral to the three later A.2 genomes, and the six viruses are members of a lineage that is further diversifying.

**Fig. 4:**
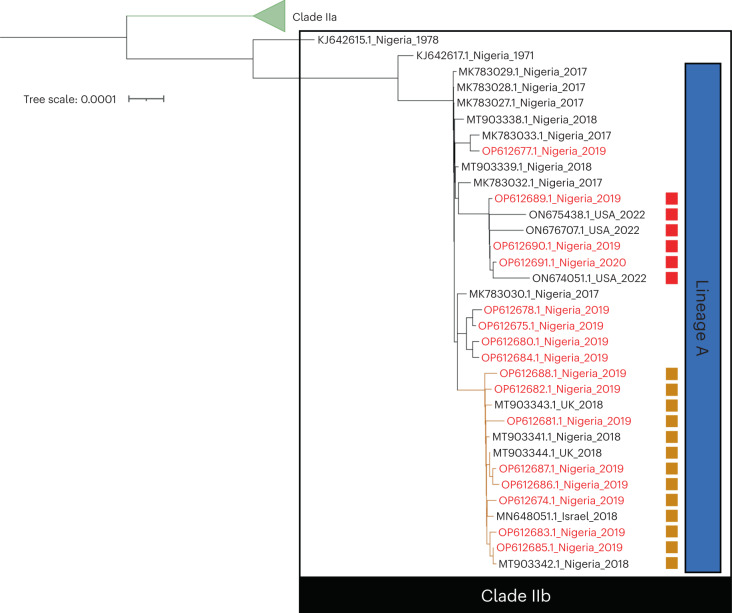
Phylogenetic tree of lineage A MPXV genomes. The tree is generated using concatenated nucleotide ORF sequences extracted from complete genomes with a limited number of unknown bases in their sequence, after genes with any ambiguous nucleotides in their sequence have been excluded for all sequences. Genomes described in this study are highlighted in red, and those with a disrupted *A46R* gene (lineage A.2) are marked with a red square. Genomes from an additional potential lineage are marked with a brown square.

To investigate these viruses further, we undertook a more detailed analysis, using only concatenated nucleotide open reading frame (ORF) sequences from complete genomes, and excluding genomes with long runs of ambiguous nucleotides in the assembled sequences. With this alternative approach, these six viruses form a cluster on a long branch separating them from other clade IIb lineage A viruses (Fig. 4), supporting the lineage A.2 designation given to the three US isolates in the cluster by Gigante et al.^14^. In addition, using this analysis, a further cluster including viruses exported to the United Kingdom and Israel in 2018 appears to have branched away and may potentially be regarded as a distinct lineage. Although this cluster does not have an identifying characteristic like the A.2 lineage (see below), taken with the A.2 and B.1 lineages it is a clear indication that multiple lineages of MPXV have achieved sustained human-to-human transmission from the presumed single zoonotic event, and have coexisted in humans over a period of several years.

### Gene disruption in lineage A2

The three lineage A.2 genomes presented here (OP612690, OP612689 and OP612691) have an identical nonsense mutation in the *A46R* gene (Copenhagen nomenclature^22^) that is compatible with APOBEC3 activity (Supplementary Table 1) and is not found in any of the other isolates described here. These three isolates were collected between December 2019 and January 2020. OP612690 and OP612689 were from individuals in Imo and Rivers states, respectively, and are adjacent on the phylogenetic tree. Geographic metadata are missing for OP612691, but the genome is in the same phylogenetic cluster with OP612690 and OP612689. Importantly, we have found the identical mutation in the three A.2 isolates from the US ON676707 (July 2021), ON674051 and ON675438 (May 2022), and these three, which are independent of the B.1 lineage^14^, form a contiguous cluster with OP612690, OP612689 and OP612691 on the phylogenetic tree (Figs. 1a and 4). The *A46R* gene is highly conserved between MPXV and the vaccinia virus (VACV), and encodes a Toll–interleukin (IL)-1-receptor (TIR) homolog. The A46 protein interferes with IL-1-dependent signal transduction and activation of nuclear factor-κB and type I interferon responses, and deletion mutants in VACV are attenuated for virulence in mice^23,24^. The presence of an identical APOBEC3-style mutation in all six lineage A.2 genomes suggests it is not independent and, coupled with their isolation over a period from December 2019 to May 2022, that this A46 disruption variant is actively circulating among human hosts. For many SNPs, this might mean they are neutral mutations. However, a mutation that disrupts an immune modulator protein is not expected to be neutral in vivo; thus, there is a possibility that the disruption of *A46R* has adaptive value for the virus. This is worthy of experimental analysis to assess potential phenotypic effects.

MPXV encodes a plethora of proteins that have been shown in other OPXVs to modulate innate immunity, including several that interfere with IL-1 responses. In experimental systems, disrupting these genes is often attenuating in laboratory mice. However, disruptions are also seen in natural systems, most notably in the variola virus, the causative agent of smallpox, where sequencing studies have shown that several such genes are disrupted, including genes that antagonize the IL-1 signaling pathway (*A52R*) and the soluble IL-1β binding protein (*B16R*; Copenhagen nomenclature^22^)^25,26^. In addition, it is known that OPXVs adapt to new hosts through a process of gene loss, analogous to the disruption of *A46R* (ref. ^27^). The fact that an identical mutation is present in all six lineage A.2 genomes suggests that this mutation arose once in the ancestor of this lineage and has persisted as the lineage spread for over 30 months over two continents.

## Discussion

Human mpox continues to be reported from Nigeria^28^. It is also detected more widely in Africa, including from non-endemic African countries^29,30^. The future spread of MPXV in humans after the advent of lineage B.1 will provide an important resource for understanding how, or if, it is adapting to better exist as a human virus, and if the success of lineage B.1 is primarily a founder effect or represents an adaptive breakthrough. The data reported here, with more genomes than have previously been recovered from the time between the initial zoonotic event(s) and January 2020, provide an essential baseline for any future analysis that looks at the evolution of human mpox both in Nigeria and elsewhere. We observed mutations in the targets for the two antivirals licensed for smallpox and also used against mpox. None of the changes match previously described resistance mutations^31,32^, and we do not expect that efficacy of the drugs will be affected by these specific mutations, given that neither drug was in use in Nigeria at the time, and their broad activity for OPXVs. However, it demonstrates that mutations are detectable at the population level in these drug targets even in the absence of selective pressure from application of the drugs themselves. The information from the sequences reported here will be vital both for our understanding of the evolution and origin of lineage B viruses, and future lineages that may potentially arise from lineage A.

The possibility that APOBEC3 enzymes may be active on MPXV in humans is an important consideration for our understanding of the potential for MPXV to adapt in humans. APOBEC3 enzymes have antiviral functions against HIV^33^ and have been shown to be active on MPXV in APOBEC3 transfected cells^34^. TC dinucleotides recognized by APOBEC3 are common in the MPXV genome. However, the frequency of TC-to-TT changes and the increase over time suggest the process is relatively inefficient, with only a small number of mutations arising in a replication cycle; and the rate of accumulation may be below the threshold required for antiviral activity. However, a recent study suggests that APOBEC3-style mutations in MPXV arise with greater frequency in patients than is indicated from recovered viable viruses, and that deleterious mutations may be suppressed^34^. The distribution of APOBEC3-style mutations in the various genomes reported here and elsewhere suggests the specific TC dinucleotides mutated to TT in a replication cycle is effectively random. Taken with the reported suppression of harmful mutations, the continuous and measurable divergence with respect to APOBEC3-style mutations as mpox spreads in human populations will generate variation on which natural selection can work to adapt the virus to its new host.

The description of three new members of lineage A.2, its persistence in humans for at least 30 months, and a potentially lineage-defining mutation in a known virus immunomodulatory gene supports the designation of A.2 as a distinct lineage. Whether the *A46R* gene disruption conferred adaptive value in the transmission of lineage A.2 remains unknown. Such a disruption is consistent with the evolution of variola virus in humans where the variola *A52R* and *B16R* immunomodulator genes are disrupted^26,27^, although *A46R* itself is intact in variola. The dynamics with which variants emerge and displace parents in a natural system are not understood for poxviruses, but viruses transmitted by sustained skin-to-skin contact will have different transmission dynamics to respiratory infections such as severe acute respiratory syndrome coronavirus disease 2, and this is likely to delay the spread of variants. To date, variants disrupted in known immune modulators as described here for lineage A.2 are not present in viruses descending from lineage B.1. However, our results demonstrate that MPXV has the capacity to diverge into subpopulations with higher variability than that currently observed across lineage B.1. Lineage A is ancestral to lineage B.1, and the baseline variation in lineage A is thus anticipated to be greater than the variation in lineage B.1. Although lineage B.1 has significantly more mutations relative to the presumed ancestor than any known lineage A virus, this may be a result of lineage B.1 entering a global demographic that facilitates rapid transmission and associated APOBEC3-style mutations. Therefore, we should be aware of the possibility of the genesis of variants with adaptive value in lineage A, especially if MSM sexual contact plays a lesser role in the sustained human transmission observed in West Africa than in the global expansion success of B.1. Of critical importance for public health considerations, the level of variability observed in West Africa also suggests that even if lineage B.1 is eliminated, lineage A MPXV will continue to evolve if its human transmission chain is not broken; and there is no a priori reason to expect transmission of future variants will be limited to close or extended skin-to-skin contact.

### Limitations of the study

This study is based on 18 human MPXV genomes from Nigeria. Although the phylogenetic trees in Figs. 1 and 4 indicate they are diverse within the context of other lineage A viruses, there is no doubt that a greater number of sequences would give a more definitive picture of the diversification and evolution of MPXV in humans in the run up to the emergence of lineage B.1. However, as far as we are aware, these are the only available samples for sequencing from Jan 2019 to Jan 2020, and the information they provide is thus invaluable for our understanding of the evolution of MPXV.

There are few if any human mpox genomes available from Nigeria from between January 2020 and May 2022, which is a major gap in our knowledge of how the 2022 outbreak virus emerged. However, if genomes from between January 2020 and 2022 in Nigeria become available in the future, the data presented here will provide an essential bridge toward the original zoonotic event(s).

Current genome sequencing approaches often combine long-read nanopore sequencing with short-read Illumina sequencing, to provide two orthogonal approaches that collectively resolve potential sequencing errors and increase confidence in the accuracy of the sequences. In this study, only nanopore sequencing was available. However, good read depth was achieved, and apparent mutations likely to be sequencing errors (for example, in homopolymeric regions) are listed as such in Table 1 and were not used for generating phylogenetic trees. In addition, the inclusion of a phylogenetic tree based on concatenated nucleotide ORF sequences (Fig. 4), and the occurrence of particular APOBEC3-style mutations in multiple genomes, increases confidence in the analysis.

**Table 1:** MPXV genomes described in this study

Genome identifier	Genome length (bp)	APOBEC3 count versus MT903345	APOBEC3 count versus KJ642617	Gender	Date of isolation	Location (state)
OP612674	197,409	6	29	Male	2019	Bayelsa
OP612675	197,128	12	22	Male	2019	Bayelsa
OP612676	194,651	10	20	Female	2019	Bayelsa
OP612677	197,533	20	31	Male	2019	Rivers
OP612678	197,628	13	23	Male	2019	Rivers
OP612679	197,356	8	33	Male	2019	Delta
OP612680	195,014	12	23	Male	2019	Lagos
OP612681	197,207	7	29	Female	2019	Bayelsa
OP612682	197,160	6	28	Male	2019	Bayelsa
OP612683	197,252	2	26	Male	2019	Delta
OP612684	197,704	12	23	Male	2019	Lagos
OP612685	197,248	2	24	Male	2019	Delta
OP612686	197,125	8	33	Female	2019	Delta
OP612687	197,538	10	35	Male	2019	Enugu
OP612688	197,097	11	33	Male	2019	Oyo
OP612689	197,177	14	24	Male	2019	Rivers
OP612690	197,372	13	24	Male	2019	Imo
OP612691	197,386	16	26	No data	2020	No data

## Conclusion

So far there is no indication that different clade IIb MPXV lineages have differential pathogenicity. However, the scale of the global lineage B.1 outbreak demonstrates the potential for MPXV to spread through human populations, albeit so far in a restricted demographic. There would be a clear advantage to the virus to improve transmission outside the MSM populations that have predominantly suffered from lineage B.1. The continuing transmission of lineage A in Nigeria, coupled with the greater variation in lineage A than in lineage B.1 and the demonstration that lineage A.2 has acquired a disruption in a virus immunomodulator gene, suggest that lineage A may have enduring potential to give rise to widespread human disease agents. It is important to address the ongoing transmission of lineage A MPXVs in Nigeria, as the best way to prevent a successor to lineage B.1. It is also important to undertake laboratory analysis of divergent lineages to understand whether phenotypic divergence is occurring, and the potential for new lineages with defined features such as specific gene disruptions to have increased pathogenicity and/or transmissibility.

## Methods

### Sample collection and nucleic acid extraction

Twenty lesion swab samples archived at Nigeria CDC from patients with confirmed mpox were selected for sequencing. Samples were selected based on sample Ct values on PCR and collection date to give as wide a temporal range as possible at the time. A Ct value of between 14.90 (OP612675) and 19.98 (OP612677; mean, 17.82) was recorded for samples. Before extraction, background host DNA was removed with an adapted nuclease treatment^35^, incubating with HL-SAN Nuclease (ArticZymes) at a final concentration of 1 U µl^−1^, for 15 min at 37 °C. Samples were then inactivated and extracted using QIAamp DNA Mini Kit (Qiagen), per the manufacturer’s instructions. Only 18 samples returned complete genome sequences. The two samples that did not return complete genomes (see below) were not included in the analysis. Samples and metadata were anonymized to prevent any identification of individuals. A retrospective ethical waiver for use of clinical samples was obtained from the National Health Research Ethics Committee of Nigeria (ref. NHREC/01/01/2007-19 /03/2019).

### Sequencing

To produce sufficient quantities of material to sequence, DNA samples were tagged, randomly primed and amplified using a modified sequence-independent amplification^36–38^. A total of five sequencing runs using the Oxford Nanopore Technologies (ONT) MinION sequencer and R9.41 flow cells were carried out, with each containing five mpox samples. Sequencing libraries for each were prepared using the ONT Native barcoding expansion (EXP-NDB103) with the ligation sequencing kit (SQK-LSK109) and a modified one-pot protocol^39^.

### Bioinformatics

Fastq reads were basecalled and de-multiplexed using guppy (version 3.6; ONT), with reads under a *q*-score of 7 being discarded. Following this, reads equal and above 1,000 bp were assembled using Canu (version 2.1.1) (ref. ^40^) with generated Contigs being analyzed in BLASTn (version 2.12.0) (ref. ^41^) to select a suitable mpox reference sequence for sequence read alignment. For cases where Canu failed to produce any mpox contigs, the NCBI RefSeq MPXV sequence (NC_003310) was used as a reference.

The Medaka (version 1.2.2) program (https://github.com/nanoporetech/medaka/), using the medaka consensus command and the r941_min_high_g360 model, was used to generate a polished consensus sequence. Multiple rounds of polishing were performed to improve the consensus and close gaps while incorporating INDELs. Following the final round of Medaka polishing, BEDtools (version 2.27.1) (ref. ^42^) was used to ascertain coverage across the sequenced genome. Bases with a read depth of <10 were regarded as indeterminate and replaced with ‘N’ using an in-house R script (Supplementary Table 6).

Following ONT MinION sequencing, between 5.63 × 10^4^ (OP612687) and 3.60 × 10^6^ (OP612691) fastq reads (mean, 1.36 × 10^6^) above a *q*-score of 7 were generated. Following alignment to a respective reference sequence, low sequence coverage was identified for two samples (MPXV/100/19, 0.1%; and MPXV/050/19, 50.20%). These samples were removed from all downstream analysis. For the remaining 18 samples, between 90.76% (OP612676) and 100% (OP612690 and OP612691; mean, 99.19%) of the genome was generated with an average sequence read coverage of 1,475).

Sequences were aligned with 337 previously sequenced mpox isolates using MAFFT^43^ (version 7.453; maximum iterations, 15) before the first 14,619 bp and last 17,148 bp of all sequences were trimmed. Following this, a maximum likelihood phylogenetic tree showing the evolutionary relationship between aligned MPXV isolates was generated using IQ-Tree^44^ (version 2.1.3; MFP model selected; replicates for bootstrap, 1,000). Finally, all output genomes were compared against a sequence characterized clade II isolate (KJ642617) to characterize the SNP and INDEL differences. KJ642617 is from the clade II, isolated from a human case in Nigeria in 1971.

We also generated a whole-genome tree based on coding regions by identifying a smaller set of high-quality genomes with a small number of unknown bases in their sequence. We reannotated them with Splign^45^ (version 2.1.0), using the annotation of ON954773 as a starting point. mRNA sequences still present in all genomes after removal of ambiguous/unknown nucleotides (OPG024, OPG027, OPG029, OPG030, OPG031, OPG034, OPG035, OPG036, OPG037, OPG038, OPG039, OPG040, OPG042, OPG043, OPG044, OPG045, OPG046, OPG047, OPG048, OPG049, OPG050, OPG051, OPG052, OPG053, OPG054, OPG055, OPG056, OPG057, OPG058, OPG059, OPG060, OPG061, OPG062, OPG065, OPG066, OPG069, OPG070, OPG073, OPG075, OPG076, OPG077, OPG079, OPG081, OPG082, OPG083, OPG085, OPG086, OPG087, OPG089, OPG090, OPG091, OPG092, OPG093, OPG094, OPG095, OPG096, OPG097, OPG098, OPG099, OPG101, OPG102, OPG104, OPG105, OPG106, OPG107, OPG108, OPG109, OPG110, OPG111, OPG112, OPG113, OPG114, OPG115, OPG116, OPG118, OPG119, OPG120, OPG121, OPG122, OPG123, OPG124, OPG125, OPG126, OPG127, OPG128, OPG129, OPG130, OPG131, OPG132, OPG133, OPG134, OPG137, OPG138, OPG139, OPG140, OPG141, OPG142, OPG143, OPG144, OPG146, OPG147, OPG148, OPG149, OPG150, OPG151, OPG154, OPG155, OPG156, OPG157, OPG158, OPG159, OPG160, OPG161, OPG162, OPG163, OPG164, OPG165, OPG166, OPG167, OPG170, OPG171, OPG173, OPG174, OPG175, OPG176, OPG178, OPG180, OPG181, OPG187, OPG189, OPG190, OPG191, OPG192, OPG193, OPG195, OPG198, OPG199, OPG200, OPG205 and OPG209) were collected and concatenated for each genome. The concatenated sequences were aligned with MAFFT (version 7.508) (ref. ^43^) and, from the result, a maximum likelihood phylogenetic tree was produced with RAxML (version 8.2.12) (ref. ^46^) using a nucleotide GTR + gamma model.

To characterize any APOPBEC3-style mutations (TC > TT and GA > AA) that have recently evolved, all generated NCDC and 2022 outbreak samples were aligned to the UK_P1, UK_P2 and UK_P3 (MT903343, MT903344, MT903345) MPXV genomes, sequenced from samples collected in the United Kingdom in 2018, and separately against KJ642617. A sliding window analysis (window size, 2; step size, 1) was performed using an in-house R script (Supplementary Table 7) to identify any associated APOBEC3-style dinucleotide changes.

### Additional genomes

All genomes used in this analysis are listed in Supplementary Table 5. Genomes used for comparison with the 18 isolates described here were downloaded from GenBank, including ON676707, ON674051 and ON675438 (ref. ^14^). KJ642617 was used for comparisons because it is the earliest available sequence from Nigeria, and is assumed to be directly zoonotic and thus not multiply passaged between humans.

### Statistical analysis

Summary statistics on APOBEC3-style mutations were presented by temporal group. As APOBEC3-style mutations were not normally distributed the median, range and interquartile range were presented. A Kruskal–Wallis test was first used to determine if there were significant differences between temporal groups, followed by pairwise comparisons between temporal groups made using Dunn’s tests, with a Bonferroni correction applied to adjust for multiple testing. Statistical analysis was carried out in R^47^ using the kruskal.test function from the R base package, and the DunnTest function from the FSA package^48^.

### Reporting summary

Further information on research design is available in the Nature Portfolio Reporting Summary linked to this article.

## Online content

Any methods, additional references, Nature Portfolio reporting summaries, source data, extended data, supplementary information, acknowledgements, peer review information; details of author contributions and competing interests; and statements of data and code availability are available at 10.1038/s41591-023-02456-8.

## Supplementary information

Supplementary Information Supplementary Tables 1–7.

Reporting Summary

## Data Availability

Genomes for the 18 isolates described here are deposited in GenBank; accession numbers are listed in Table 1.
