# Supplementary material for: Distinct monkeypox virus lineages co-circulating in humans before 2022
Source: Nat Med. 2023 Sep 14;29(9):2317–24. doi: 10.1038/s41591-023-02456-8 (PMC10504077; doi:10.1038/s41591-023-02456-8)
Supplement: Supplementary file 1 — Supplementary Tables 1–7. [file 41591_2023_2456_MOESM1_ESM.pdf]

nature medicine

Article https://doi.org/10.1038/s41591-023-02456-8

Distinct monkeypox virus lineages co-circulating in humans before 2022

In the format provided by the authors and unedited

Genome position	Sample	Type	Reference sequence	Alternate sequence	Effect	Gene product
6744	OP612679	del	CT	C		
7110	OP612688	snp; APOBEC3 (possible error)	C	T		
8547	OP612675, OP612676, OP612678, OP612680, OP612684	snp; APOBEC3	C	T		
8662	OP612679	snp; APOBEC3 (possible error)	C	T		
9055	OP612674, OP612675, OP612676, OP612677, OP612678, OP612680, OP612681, OP612682, OP612683, OP612684, OP612685, OP612686, OP612687, OP612688, OP612689, OP612690, OP612691	snp	C	A		
11717	OP612674	snp; APOBEC3	C	T	R449Q	ankyrin-like protein (Copenhagen C9L)
13460	OP612689, OP612690, OP612691	snp	C	T		
13787	OP612674, OP612675, OP612676, OP612677, OP612678, OP612680, OP612681, OP612682, OP612683, OP612684, OP612685, OP612686, OP612687, OP612688, OP612689, OP612690, OP612691	snp	A	G	M548T	ankyrin-like protein (Copenhagen C9L)
16770	OP612674, OP612675, OP612676, OP612677, OP612678, OP612680, OP612681, OP612682, OP612683, OP612684, OP612685, OP612686, OP612687, OP612688, OP612689, OP612690, OP612691	del	GTCATCA	G	D155_D156(del)	hypothetical protein (Copenhagen C6L)

18480	OP612674, OP612675, OP612677, OP612678, OP612679, OP612681, OP612682, OP612683, OP612684, OP612685, OP612686, OP612687, OP612688, OP612689, OP612690, OP612691	snp; APOBEC3	G	A	L173L	hypothetical protein (Copenhagen C4L)
18960	OP612689, OP612690, OP612691	snp; APOBEC3	C	T	D13N	hypothetical protein (Copenhagen C4L)
19535	OP612674, OP612675, OP612676, OP612677, OP612678, OP612680, OP612681, OP612682, OP612683, OP612684, OP612685, OP612686, OP612687, OP612688, OP612689, OP612690, OP612691	snp; APOBEC3	C	T	S213S	hypothetical protein (Copenhagen C1L)
23139	OP612681	snp; APOBEC3	G	A	I63I	hypothetical protein (Copenhagen M2L)
23521	OP612677	snp; APOBEC3	C	T	D256N	ankyrin-like protein (Copenhagen K1L)
23732	OP612674, OP612679, OP612681, OP612682, OP612683, OP612686, OP612687, OP612688	snp; APOBEC3	C	T	S185S	ankyrin-like protein (Copenhagen K1L)
23879	OP612681	snp; APOBEC3	G	A	V136V	ankyrin-like protein (Copenhagen K1L)
25240	OP612691	snp; APOBEC3	C	T	R136Q	serine protease inhibitor-like protein SPI-3 (Copenhagen K2L)
25829	OP612679, OP612686, OP612687	snp; APOBEC3	G	A		
28332	OP612675, OP612676, OP612678	snp; APOBEC3	C	T	S25F	B15R-like protein (Copenhagen K7R)
28813	OP612674	snp; APOBEC3	G	A	I206I	hypothetical protein ### (Copenhagen F1L)
29916	OP612674, OP612675, OP612676, OP612677, OP612678, OP612680, OP612681, OP612682, OP612683, OP612684, OP612685, OP612686,	del	ACCATT	A	N483fs	kelch-like protein (Copenhagen F3L)

	OP612688, OP612689, OP612690, OP612691					
30062	OP612675, OP612676, OP612678, OP612680, OP612684	snp; APOBEC3	G	A	P436S	kelch-like protein (Copenhagen F3L)
30809	OP612689, OP612690, OP612691	snp; APOBEC3	C	T	D187N	kelch-like protein (Copenhagen F3L)
32381	OP612674, OP612675, OP612676, OP612677, OP612678, OP612680, OP612681, OP612682, OP612683, OP612684, OP612685, OP612686, OP612687, OP612688, OP612689, OP612690, OP612691	ins	T	TGAC	S313_H314(ins)R	hypothetical protein (Copenhagen F5L)
33313	OP612674, OP612675, OP612676, OP612677, OP612678, OP612680, OP612681, OP612682, OP612683, OP612684, OP612685, OP612686, OP612687, OP612688, OP612689, OP612690, OP612691	snp; APOBEC3	C	T	T3T	hypothetical protein (Copenhagen F5L)
33347	OP612681	snp; APOBEC3	G	A		
34642	OP612689, OP612690, OP612691	snp; APOBEC3	C	T	V73V	hypothetical protein (Copenhagen F9L)
34748	OP612689, OP612690, OP612691	snp; APOBEC3	C	T	G38E	hypothetical protein (Copenhagen F9L)
37379	OP612675, OP612676, OP612678, OP612680, OP612684	snp; APOBEC3	G	A	S609F	hypothetical protein (Copenhagen F12L)
38237	OP612674, OP612675, OP612676, OP612677, OP612678, OP612680, OP612681, OP612682, OP612683, OP612684, OP612685, OP612686, OP612687, OP612688, OP612689, OP612690, OP612691	snp	G	A	A323V	hypothetical protein (Copenhagen F12L)
39289	OP612679, OP612686, OP612687	snp; APOBEC3	C	T	E359E	palmytilated EEV membrane protein

						(Copenhagen F13L)
39937	OP612685	snp; APOBEC3	C	T	T143T	palmytilated EEV membrane protein (Copenhagen F13L)
40421	OP612680, OP612684	snp; APOBEC3	C	T	D62N	hypothetical protein (Copenhagen F14L)
43288	OP612675, OP612678, OP612680, OP612684	snp; APOBEC3	G	A	L191L	poly-A polymerase catalytic subunit VP55 (Copenhagen E1L)
46855	OP612678	snp; APOBEC3	G	A	F248F	bifunctional DNA-dependent RNA polymerase subunit rpo30 (Copenhagen E4L)
47065	OP612674, OP612675, OP612676, OP612677, OP612678, OP612680, OP612681, OP612682, OP612683, OP612684, OP612685, OP612686, OP612687, OP612688, OP612689, OP612690, OP612691	snp	G	A	D178D	bifunctional DNA-dependent RNA polymerase subunit rpo30 (Copenhagen E4L)
47394	OP612688	snp; APOBEC3	C	T	E69K	bifunctional DNA-dependent RNA polymerase subunit rpo30 (Copenhagen E4L)
48308	OP612674, OP612675, OP612676, OP612677, OP612678, OP612680, OP612681, OP612682, OP612683, OP612684, OP612685, OP612686, OP612687, OP612688, OP612689, OP612690, OP612691	snp	A	G		
48350	OP612675, OP612676, OP612678, OP612680, OP612684	snp; APOBEC3	G	A		
48697	OP612674, OP612675, OP612676, OP612677, OP612678, OP612680, OP612681, OP612682, OP612683, OP612684, OP612685, OP612686,	snp	C	A	T109T	hypothetical protein (Copenhagen E6R)

	OP612687, OP612688, OP612689, OP612690, OP612691					
51979	OP612677	snp; APOBEC3	C	T	R877Q	DNA polymerase (Copenhagen E9L)
53377	OP612674, OP612675, OP612676, OP612677, OP612678, OP612680, OP612681, OP612682, OP612683, OP612684, OP612685, OP612686, OP612687, OP612688, OP612689, OP612690, OP612691	snp	C	A	W411L	DNA polymerase (Copenhagen E9L)
56521	OP612677	snp; APOBEC3	C	T	E259K	hypothetical protein (Copenhagen O1L)
57138	OP612677	snp; APOBEC3	G	A	S53L	hypothetical protein (Copenhagen O1L)
62014	OP612677	snp; APOBEC3	G	A	L59L	large subunit of ribonucleotide reductase protein (Copenhagen I4L)
63434	OP612680	snp; APOBEC3	C	T	L63L	hypothetical protein (Copenhagen I6L)
63971	OP612680, OP612684	snp; APOBEC3	C	T	E306K	viral core Cysteine proteinase (Copenhagen I7L)
66750	OP612674, OP612675, OP612676, OP612677, OP612678, OP612681, OP612682, OP612683, OP612684, OP612685, OP612686, OP612687, OP612688, OP612689, OP612690, OP612691	snp; APOBEC3	G	A	R620Q	bifunctional DNA/RNA-helicase/DExH-NPH-II (Copenhagen I8R)
69187	OP612682	snp; APOBEC3	C	T	S54F	late transcription elongation factor (Copenhagen G2R)
69848	OP612677	snp; APOBEC3	G	A	F62F	hypothetical protein (Copenhagen G4L)
72532	OP612674, OP612679, OP612681, OP612682, OP612683, OP612686, OP612687, OP612688	snp; APOBEC3	C	T	D196N	virion structural protein (Copenhagen G7L)

74202	OP612677	snp	G	A	A85T	Myristylprotein (Copenhagen G9R)
76137	OP612674	snp	G	A	S303S	hypothetical protein (Copenhagen L3L)
76248	OP612674, OP612675, OP612677, OP612678, OP612680, OP612682, OP612683, OP612684, OP612686, OP612687, OP612688, OP612690, OP612691	Del (possible error)	CT	C	K266fs	hypothetical protein (Copenhagen L3L)
77805	OP612681	snp; APOBEC3	G	A	D246N	core protein vp8 (Copenhagen L4R)
77896	OP612688	snp; APOBEC3 (possible error)	C	T	S21F	putative membrane protein (Copenhagen L5R)
79708	OP612679	snp; APOBEC3	G	A	D152N	bifunctional subunit of multifunctional poly-A polymerase (Copenhagen J3R)
80269	OP612689, OP612690, OP612691	snp; APOBEC3	G	A	L33L	DNA-dependent RNA polymerase subunit rpo22 (Copenhagen J4R)
81156	OP612674, OP612675, OP612676, OP612677, OP612678, OP612680, OP612681, OP612682, OP612683, OP612684, OP612685, OP612686, OP612687, OP612688, OP612689, OP612690, OP612691	snp	C	T	A12T	late 16 kDa putative membrane protein (Copenhagen J5L)
81445	OP612686	snp; APOBEC3	G	A	K50K	DNA-dependent RNA polymerase subunit rpo147 (Copenhagen J6R)
81524	OP612679	snp; APOBEC3	G	A	E77K	DNA-dependent RNA polymerase subunit rpo147 (Copenhagen J6R)
81693	OP612683	snp	G	A	S133N	DNA-dependent RNA polymerase subunit rpo147 (Copenhagen J6R)
83496	OP612674, OP612679, OP612681, OP612682, OP612683, OP612686, OP612687, OP612688	snp; APOBEC3	C	T	S734L	DNA-dependent RNA polymerase subunit rpo147 (Copenhagen J6R)

83666	OP612681	snp; APOBEC3	C	T	L791L	DNA-dependent RNA polymerase subunit rpo147 (Copenhagen J6R)
84178	OP612677	snp	C	T	F961F	DNA-dependent RNA polymerase subunit rpo147 (Copenhagen J6R)
84586	OP612679	snp; APOBEC3	G	A	K1097K	DNA-dependent RNA polymerase subunit rpo147 (Copenhagen J6R)
87197	OP612675, OP612676, OP612678	snp	C	T	V11V	IMV heparin binding surface protein (Copenhagen H3L)
87400	OP612674, OP612679, OP612681, OP612682, OP612683, OP612686, OP612687, OP612688	snp; APOBEC3	G	A	H740Y	RAP94 (Copenhagen H4L)
87467	OP612674, OP612679, OP612681, OP612682, OP612683, OP612686, OP612687, OP612688	snp; APOBEC3	G	A	F717F	RAP94 (Copenhagen H4L)
88810	OP612686	snp; APOBEC3	G	A	R270C	RAP94 (Copenhagen H4L)
88906	OP612679	snp; APOBEC3 (possible error)	C	T	E238K	RAP94 (Copenhagen H4L)
89269	OP612677, OP612678, OP612679, OP612684, OP612687	del (possible error)	TA	T	F116fs	RAP94 (Copenhagen H4L)
90499	OP612691	snp; APOBEC3	C	T	S22L	topoisomerase type IB (Copenhagen H6R)
91115	OP612681	snp; APOBEC3	C	T	V227V	topoisomerase type IB (Copenhagen H6R)
91898	OP612674, OP612679, OP612681, OP612682, OP612683, OP612686, OP612687, OP612688	snp; APOBEC3	G	A		
92223	OP612677	snp; APOBEC3	C	T	S108L	bifunctional large subunit of mRNA capping enzyme protein (Copenhagen D1R)
92686	OP612674, OP612675, OP612676, OP612677, OP612678, OP612680, OP612681, OP612682, OP612683, OP612684,	snp	T	C	D262D	bifunctional large subunit of mRNA capping enzyme protein (Copenhagen D1R)

	OP612685, OP612686, OP612687, OP612688, OP612689, OP612690, OP612691					
93000	OP612684	snp; APOBEC3	C	T	S367F	bifunctional large subunit of mRNA capping enzyme protein (Copenhagen D1R)
93557	OP612689, OP612690, OP612691	snp; APOBEC3	G	A	E553K	bifunctional large subunit of mRNA capping enzyme protein (Copenhagen D1R)
95010	OP612674	snp; APOBEC3	G	A	E61K	virion core protein (Copenhagen D3R)
97011	OP612677	snp; APOBEC3	G	A	D265N	NTPase (Copenhagen D5R)
98658	OP612674, OP612675, OP612676, OP612677, OP612678, OP612680, OP612681, OP612682, OP612683, OP612684, OP612685, OP612686, OP612687, OP612688, OP612689, OP612690, OP612691	ins	A	AG		
98896	OP612674, OP612675, OP612676, OP612677, OP612678, OP612680, OP612681, OP612682, OP612683, OP612684, OP612685, OP612686, OP612687, OP612688, OP612689, OP612690, OP612691	snp	T	C		
100731	OP612674, OP612675, OP612677, OP612678, OP612679, OP612681, OP612682, OP612683, OP612684, OP612685, OP612686, OP612687, OP612688, OP612689, OP612690, OP612691	snp	C	T	G59G	DNA-dependent RNA polymerase subunit rpo18 (Copenhagen D7R)
100811	OP612679	snp; APOBEC3	G	A	R86K	DNA-dependent RNA polymerase subunit rpo18

						(Copenhagen D7R)
101140	OP612677	snp	C	T	E260K	IMV membrane protein (Copenhagen D8L)
104917	OP612680	snp	T	C	D108G	bifunctional ATPase/nucleoside triphosphate phosphohydrolase-I (Copenhagen D11L)
105295	OP612674, OP612675, OP612676, OP612677, OP612678, OP612680, OP612681, OP612682, OP612683, OP612684, OP612685, OP612686, OP612687, OP612688, OP612689, OP612690, OP612691	snp	T	C	R281R	bifunctional small subunit of mRNA capping enzyme protein (Copenhagen D12L)
114010	OP612675, OP612676, OP612678, OP612680, OP612684	snp; APOBEC3	C	T	G619E	82 kDa large subunit of early gene transcription factor VETF (Copenhagen A7L)
114828	OP612689, OP612690, OP612691	snp	G	A	A346A	82 kDa large subunit of early gene transcription factor VETF (Copenhagen A7L)
119465	OP612674, OP612679, OP612681, OP612682, OP612683, OP612686, OP612687, OP612688	snp; APOBEC3	C	T	D98N	precursor p4a of core protein 4a (Copenhagen A10L)
119887	OP612674, OP612675, OP612676, OP612677, OP612678, OP612680, OP612681, OP612682, OP612683, OP612684, OP612685, OP612686, OP612687, OP612688, OP612689, OP612690, OP612691	snp	C	T	T39T	hypothetical protein (Copenhagen A11R)
121284	OP612680, OP612681, OP612684, OP612689	del (possible error)	AT	A	N6fs	core protein (Copenhagen A12L)
121489	OP612674, OP612679, OP612681, OP612682, OP612683, OP612686, OP612687, OP612688	snp	C	T	A17T	IMV membrane protein (Copenhagen A13L)

121623	OP612675, OP612676, OP612678	snp; APOBEC3	G	A		
123169	OP612677	snp; APOBEC3	C	T	E106K	soluble myristylprotein (Copenhagen A16L)
124200	OP612689, OP612690, OP612691	snp; APOBEC3	G	A	D29N	DNA helicase (Copenhagen A18R)
124299	OP612686, OP612687	snp; APOBEC3	G	A	E62K	DNA helicase (Copenhagen A18R)
125418	OP612674, OP612679, OP612681, OP612682, OP612683, OP612686, OP612687, OP612688	snp; APOBEC3 (possible error)	G	A	E435K	DNA helicase (Copenhagen A18R)
125661	OP612677	snp; APOBEC3	C	T	D50N	hypothetical protein (Copenhagen A19L)
128245	OP612674, OP612675, OP612676, OP612677, OP612678, OP612680, OP612681, OP612682, OP612683, OP612684, OP612685, OP612686, OP612687, OP612688, OP612689, OP612690, OP612691	snp; APOBEC3	G	A	D100N	45 kDa large subunit of intermediate gene transcription factor VITF-3 (Copenhagen A23R)
128343	OP612677	snp	C	T	V132V	45 kDa large subunit of intermediate gene transcription factor VITF-3 (Copenhagen A23R)
128867	OP612686, OP612687	snp; APOBEC3	C	T	S307L	45 kDa large subunit of intermediate gene transcription factor VITF-3 (Copenhagen A23R)
129931	OP612674, OP612675, OP612676, OP612677, OP612678, OP612680, OP612681, OP612682, OP612683, OP612684, OP612685, OP612686, OP612687, OP612688, OP612689, OP612690, OP612691	snp; APOBEC3	G	A	G280D	DNA-dependent RNA polymerase subunit rpo132 (Copenhagen A24R)
132689	OP612691	snp; APOBEC3	C	T		
133263	OP612680	del (possible error)	TTTTTTTTTC	T		

133265	OP612675, OP612676	del (possible error)	TTTTTTTC	T		
133267	OP612686, OP612687, OP612689	del (possible error)	TTTTTC	T		
133268	OP612677, OP612682	del (possible error)	TTTTC	T		
133269	OP612678, OP612681, OP612684, OP612691	del (possible error)	TTTC	T		
133270	OP612685, OP612688	del (possible error)	TTC	T		
133271	OP612690	del (possible error)	TC	T		
133272	OP612679	del (possible error)	C	T		
133272	OP612683	del (possible error)	C	TTT		
133272	OP612674	del (possible error)	C	TTTT		
133318	OP612674, OP612675, OP612676, OP612677, OP612678, OP612680, OP612681, OP612682, OP612683, OP612684, OP612685, OP612686, OP612687, OP612688, OP612689, OP612690, OP612691	snp	C	T		
133336	OP612686, OP612687	del	GCAATCTTTC T	G		
134251	OP612677	snp; APOBEC3	C	T	R672K	cowpox A-type inclusion protein (Copenhagen A26L)
136733	OP612674, OP612675, OP612676, OP612678, OP612679, OP612681, OP612682, OP612683, OP612684, OP612685, OP612686, OP612689, OP612690, OP612691	Complex (possible error)	CATNATCATC	TATGAT	D370fs	cowpox A-type inclusion protein (Copenhagen A26L)
136736	OP612687	Complex (possible error)	NATC	TATG	missense_variant c.1111_1114delGATTinsCATA p.D???371HN	cowpox A-type inclusion protein (Copenhagen A26L)
137809	OP612674, OP612675, OP612676, OP612677,	snp	G	A	T14M	cowpox A-type inclusion protein

	OP612678, OP612680, OP612681, OP612682, OP612683, OP612684, OP612685, OP612686, OP612687, OP612688, OP612689, OP612690, OP612691					(Copenhagen A26L)
138649	OP612679	del (possible error)	TA	T	F8fs	IMV surface protein (Copenhagen A28L)
138753	OP612681	snp	G	A	T280I	DNA-dependent RNA polymerase rpo35 (Copenhagen A29L)
138973	OP612679	snp; APOBEC3	C	T	D207N	DNA-dependent RNA polymerase rpo35 (Copenhagen A29L)
140286	OP612674, OP612675, OP612676, OP612677, OP612678, OP612680, OP612681, OP612682, OP612683, OP612684, OP612685, OP612686, OP612687, OP612688, OP612689, OP612690, OP612691	ins	A	AATAACAATT	N123_C124(ins)NYN	hypothetical protein (Copenhagen A31R)
141473	OP612674, OP612675, OP612676, OP612677, OP612678, OP612680, OP612681, OP612682, OP612683, OP612684, OP612685, OP612686, OP612687, OP612688, OP612689, OP612690, OP612691	snp; APOBEC3	G	A	E67K	bifunctional EEV membrane phosphoglycoprotein (Copenhagen A33R)
141537	OP612674, OP612675, OP612676, OP612677, OP612678, OP612680, OP612681, OP612682, OP612683, OP612684, OP612685, OP612686, OP612687, OP612688, OP612689, OP612690, OP612691	snp	C	T	A88V	bifunctional EEV membrane phosphoglycoprotein (Copenhagen A33R)
141930	OP612683	snp; APOBEC3	C	T	L36L	EEV glycoprotein (Copenhagen A34R)

142623	OP612674, OP612675, OP612676, OP612677, OP612678, OP612680, OP612681, OP612682, OP612683, OP612684, OP612685, OP612686, OP612687, OP612688, OP612689, OP612690, OP612691	snp	C	A	T83T	hypothetical protein (Copenhagen A35R)
144018	OP612688	snp; APOBEC3	C	T	I110I	hypothetical protein (Copenhagen A37R)
144832	OP612678	snp; APOBEC3	G	A	S250F	CD47-like putative membrane protein (Copenhagen A38L)
145049	OP612689	snp; APOBEC3	G	A	P178S	CD47-like putative membrane protein (Copenhagen A38L)
146225	OP612677	snp; APOBEC3	C	T		
146780	OP612675, OP612676, OP612678, OP612680, OP612684	snp; APOBEC3	C	T	E58K	bifunctional secreted glycoprotein (Copenhagen A41L)
146860	OP612674, OP612675, OP612676, OP612677, OP612678, OP612680, OP612681, OP612682, OP612683, OP612684, OP612685, OP612686, OP612687, OP612688, OP612689, OP612690, OP612691,	snp	T	C	D31G	bifunctional secreted glycoprotein (Copenhagen A41L)
147034	OP612674, OP612675, OP612677, OP612678, OP612680, OP612684, OP612686, OP612688, OP612689, OP612690, OP612691	ins	A	ATATTTTATATTTTATATTT		
148566	OP612674, OP612679, OP612681, OP612682, OP612683, OP612686, OP612687, OP612688	snp; APOBEC3	G	A	I328I	bifunctional hydroxysteroid dehydrogenase (Copenhagen A44L)
148682	OP612689, OP612690, OP612691	del	TCATATCA	T	N287fs	bifunctional hydroxysteroid dehydrogenase (Copenhagen A44L)

149419	OP612689, OP612690, OP612691		G	A	S44L	bifunctional hydroxysteroid dehydrogenase (Copenhagen A44L)
149898	OP612674, OP612675, OP612676, OP612677, OP612678, OP612680, OP612681, OP612682, OP612683, OP612684, OP612685, OP612686, OP612687, OP612688, OP612689, OP612690, OP612691	snp	A	G	A101A	Cu-Zn superoxide dismutase-like protein (Copenhagen A45R)
150026	OP612689, OP612690, OP612691	snp; APOBEC3	C	T	Q22*	Toll/IL1-receptor-like protein (Copenhagen A46R)
150032	OP612674, OP612675, OP612676, OP612677, OP612678, OP612680, OP612681, OP612682, OP612683, OP612684, OP612685, OP612686, OP612687, OP612688, OP612689, OP612690, OP612691	snp	A	G	N24D	Toll/IL1-receptor-like protein (Copenhagen A46R)
150560	OP612682	snp	C	T	L200L	Toll/IL1-receptor-like protein (Copenhagen A46R)
150985	OP612677	snp; APOBEC3	C	T		
151204	OP612677	snp; APOBEC3	C	T		
151459	OP612677	snp; APOBEC3	C	T		
151775	OP612675, OP612676	snp	T	G	F38C	thymidylate kinase (Copenhagen A48R)
152083	OP612683, OP612685	snp; APOBEC3	G	A	E141K	thymidylate kinase (Copenhagen A48R)
152585	OP612688	snp; APOBEC3	G	A		
154169	OP612689, OP612690, OP612691	snp; APOBEC3	G	A	D442N	DNA ligase (Copenhagen A50R)
156917	OP612676	del (possible error)	CA	C		
158319	OP612674, OP612675, OP612676, OP612677, OP612678, OP612680,	ins	T	TA		

	OP612681, OP612682, OP612683, OP612684, OP612685, OP612686, OP612687, OP612688, OP612689, OP612690, OP612691					
160327	OP612674, OP612675, OP612676, OP612677, OP612678, OP612680, OP612681, OP612682, OP612683, OP612684, OP612685, OP612686, OP612687, OP612688, OP612689, OP612690, OP612691	snp	T	G		
160981	OP612676	Ins (possible error)	G	GA	S72fs	ser/thr kinase (Copenhagen B1R)
161005	OP612674, OP612675, OP612676, OP612677, OP612678, OP612680, OP612681, OP612682, OP612683, OP612684, OP612685, OP612686, OP612687, OP612688, OP612689, OP612690, OP612691	snp	T	C	H77H	ser/thr kinase (Copenhagen B1R)
161020	OP612674, OP612675, OP612676, OP612677, OP612678, OP612680, OP612681, OP612682, OP612683, OP612684, OP612685, OP612686, OP612687, OP612688, OP612689, OP612690, OP612691	snp	G	A	T82T	ser/thr kinase (Copenhagen B1R)
161769	OP612679	snp; APOBEC3	C	T	F9F	hypothetical protein (Copenhagen B2R)
162106	OP612689, OP612690, OP612691	snp; APOBEC3	C	T	H122Y	hypothetical protein (Copenhagen B2R)
162396	OP612680, OP612684, OP612686, OP612687	snp; APOBEC3	G	A	L218L	hypothetical protein (Copenhagen B2R)
163342	OP612674, OP612675, OP612677, OP612678, OP612679, OP612681,	del	TTAAC	T		

	OP612682, OP612683, OP612684, OP612685, OP612686, OP612687, OP612688, OP612689, OP612690, OP612691					
164989	OP612674, OP612679, OP612681, OP612682, OP612683, OP612686, OP612687, OP612688	snp; APOBEC3	C	T	L500L	ankyrin-like protein (Copenhagen B4R)
165845	OP612678	snp	G	A	P189P	EEV type-I membrane glycoprotein (Copenhagen B5R)
165939	OP612689, OP612690, OP612691	snp; APOBEC3	C	T	P221S	EEV type-I membrane glycoprotein (Copenhagen B5R)
166811	OP612674, OP612675, OP612676, OP612677, OP612678, OP612680, OP612681, OP612682, OP612683, OP612684, OP612685, OP612686, OP612687, OP612688, OP612689, OP612690, OP612691	ins	A	ATT	Y166fs	ankyrin-like protein (Copenhagen B6R)
166941	OP612688	snp; APOBEC3	C	T	S19L	bifunctional 21 kDa precursor protein of 18 kDa membrane protein (Copenhagen B7R)
167811	OP612674, OP612675, OP612676, OP612677, OP612678, OP612680, OP612681, OP612682, OP612683, OP612684, OP612685, OP612686, OP612687, OP612688, OP612689, OP612690, OP612691	snp	G	T	R108I	soluble interferon-gamma receptor-like protein (Copenhagen B8R)
168275	OP612674, OP612679, OP612681, OP612682, OP612683, OP612686, OP612687, OP612688	snp; APOBEC3 (possible error)	C	T	L263F	soluble interferon-gamma receptor-like protein (Copenhagen B8R)
168360	OP612689, OP612690, OP612691	snp	G	A		

168520	OP612687	snp; APOBEC3	G	A	E46K	6 kDa intracellular viral protein (Copenhagen B9R)
169876	OP612674, OP612675, OP612677, OP612678, OP612679, OP612681, OP612683, OP612684, OP612685, OP612686, OP612689, OP612690, OP612691	ins	T	TCAGATA	T32_D33dup	hypothetical protein (Copenhagen B11R)
171045	OP612689, OP612690, OP612691	ins	C	CT		
171824	OP612674, OP612675, OP612676, OP612677, OP612678, OP612680, OP612681, OP612682, OP612683, OP612684, OP612685, OP612686, OP612687, OP612688, OP612689, OP612690, OP612691,	snp; APOBEC3	G	A	E230K	bifunctional SPI-2/CrmA protein/IL-1 convertase (Copenhagen B14R)
174703	OP612674, OP612675, OP612676, OP612677, OP612678, OP612681, OP612682, OP612683, OP612684, OP612685, OP612686, OP612687, OP612688, OP612691	ins	T	TGATGAA		
177899	OP612689, OP612690, OP612691	snp; APOBEC3	C	T	R689C	ankyrin-like protein (Copenhagen B20R)
178219	OP612674, OP612675, OP612676, OP612677, OP612678, OP612681, OP612682, OP612683, OP612684, OP612685, OP612686, OP612687, OP612688, OP612689, OP612690, OP612691	del	GTTT	G		
178605	OP612674, OP612675, OP612677, OP612678, OP612679, OP612681, OP612682, OP612683, OP612684, OP612685, OP612686, OP612687,	snp; APOBEC3	G	A		

	OP612688, OP612689, OP612690, OP612691					
180838	OP612684	snp; APOBEC3	G	A	D47N	hypothetical protein (Copenhagen C14L)
181427	OP612674, OP612675, OP612677, OP612678, OP612679, OP612681, OP612682, OP612683, OP612684, OP612685, OP612686, OP612687, OP612688, OP612689, OP612690, OP612691	ins	G	GA		
182377	OP612682	snp; APOBEC3	G	A	D281N	putative membrane-associated glycoprotein (Copenhagen D9R)
184218	OP612688	snp	G	T	T894T	putative membrane-associated glycoprotein (Copenhagen D9R)
184716	OP612688	snp; APOBEC3	C	T	I1060I	putative membrane-associated glycoprotein (Copenhagen D9R)
186604	OP612684	snp	G	A	E1690K	putative membrane-associated glycoprotein (Copenhagen D9R)
187253	OP612674, OP612675, OP612677, OP612682, OP612683, OP612685, OP612686, OP612687, OP612688, OP612689, OP612690, OP612691	ins	A	AT		
187608	OP612674, OP612677, OP612679, OP612681, OP612682, OP612685, OP612686, OP612687, OP612688	snp; APOBEC3	C	T		
188683	OP612674	snp; APOBEC3	C	T		
189069	OP612677	snp; APOBEC3	G	A		

Table S1. Mutations in 18 MPXV genomes isolated from humans in Nigeria between January 2019 and January 2020, relative to the 1971 zoonotic Nigeria isolate KJ642617. Mutations occurring in homopolymer sequence are labelled as “possible error”, as only nanopore sequencing was available for this study. Gene designations are given using the *Vaccinia virus* Copenhagen nomenclature[22].

Statistic \ Temporal group	2018	2019	2021	2022	2022 (Other)
**n**	2	18	2	288	2
**Minimum**	0	2	31	34	27
**Lower (25th) Quartile**	0.25	4	31.8	41	28
**Median**	0.5	8.5	32.5	42	29
**Upper (75th) Quartile**	0.75	12.8	33.2	43	30
**Maximum**	1	20	34	48	31
**Kruskall-Wallis test for 2019 vs 2022** P=8.37x10^{-14}					

Table S2. Summary statistics for APOBEC3 style mutation frequency by temporal group against a baseline of 2018 UK isolates, and statistical analysis of 2019 values vs 2022.

Statistic \ Temporal group	2017	2018	2019	2021	2022	2022 (Other)
**n**	7	10	18	2	288	2
**Minimum**	15	13	18	41	58	37
**Lower (25th) Quartile**	15.5	14.2	23	45.8	66	38.2
**Median**	16	22.5	25	50.5	67	39.5
**Upper (75th) Quartile**	20.5	25	27.5	55.2	68	40.8
**Maximum**	28	26	33	60	72	42

Table S3. Summary statistics for APOBEC3 style mutation frequency by temporal group against a baseline of 1971 Zoonotic Nigeria isolate KJ642617.

	2017	2018	2019
**2017**			
**2018**	>0.999		
**2019**	>0.999	>0.999	
**2022**	9.26e-06	1.15e-07	1.22e-11

Table S4. *P* values from Dunn's test for pairwise comparisons of APOBEC3 style mutation frequency by temporal group against a baseline of 1971 Zoonotic Nigeria isolate KJ642617. Bonferroni correction was applied to adjust for multiple testing, following Kruskal-Wallis test result indicating significant differences between groups ($p<0.001$).

Genome identifier	APOBEC3 count vs MT903345	APOBEC3 count vs KJ642617	Year of isolation	Country
KJ642617	ND	N/A	1971	Nigeria
MK783027.1	ND	15	2017	Nigeria
MK783028.1	ND	15	2017	Nigeria
MK783029.1	ND	16	2017	Nigeria
MK783030.1	ND	21	2017	Nigeria
MK783031.1	ND	16	2017	Nigeria
MK783032.1	ND	20	2017	Nigeria
MK783033.1	ND	28	2017	Nigeria
MN648051.1	ND	25	2018	Israel
OP612674	6	29	2019	Nigeria
OP612675	12	22	2019	Nigeria
OP612676	10	20	2019	Nigeria
OP612677	20	31	2019	Nigeria
OP612678	13	23	2019	Nigeria
OP612679	8	33	2019	Nigeria
OP612680	12	23	2019	Nigeria
OP612681	7	29	2019	Nigeria
OP612682	6	28	2019	Nigeria
OP612683	2	26	2019	Nigeria
OP612684	12	23	2019	Nigeria
OP612685	2	24	2019	Nigeria
OP612686	8	33	2019	Nigeria
OP612687	10	35	2019	Nigeria
OP612688	11	33	2019	Nigeria
OP612689	14	24	2019	Nigeria
OP612690	13	24	2019	Nigeria
OP612691	16	26	2020	Nigeria
MT903337.1	ND	13	2018	Nigeria
MT903338.1	ND	14	2018	Nigeria
MT903339.1	ND	14	2018	Nigeria
MT903340.1	ND	15	2018	Nigeria
MT903341.1	ND	22	2018	Nigeria
MT903342.1	ND	26	2018	Nigeria
MT903343.1	ND	23	2018	UK
MT903344.1	ND	25	2018	UK
MT903345.1	N/A	25	2018	UK
ON563414.3	41	66	2022	USA
ON568298.1	42	68	2022	Germany
ON585029.1	37	59	2022	Portugal

ON585030.1	41	65	2022	Portugal
ON585031.1	41	65	2022	Portugal
ON585032.1	41	66	2022	Portugal
ON585033.1	42	67	2022	Portugal
ON585034.1	41	66	2022	Portugal
ON585035.1	41	66	2022	Portugal
ON585036.1	41	64	2022	Portugal
ON585037.1	44	69	2022	Portugal
ON585038.1	44	69	2022	Portugal
ON595760.2	41	66	2022	Switzerland
ON602722.2	40	65	2022	France
ON609725.2	45	70	2022	Spain
ON614676.1	36	61	2022	Italy
ON615424.1	43	69	2022	Netherlands
ON619835.2	41	66	2022	UK
ON619836.2	43	68	2022	UK
ON619837.2	42	67	2022	UK
ON619838.2	43	68	2022	UK
ON622712.1	42	67	2022	Belgium
ON622713.1	43	68	2022	Belgium
ON622718.1	44	69	2022	Spain
ON622720.1	39	60	2022	Switzerland
ON622721.1	44	67	2022	Italy
ON622722.2	44	69	2022	France
ON627808.1	41	67	2022	USA
ON631241.1	41	66	2022	Slovenia
ON631963.1	43	69	2022	Australia
ON637938.1	42	67	2022	Germany
ON637939.1	43	68	2022	Germany
ON644344.1	42	67	2022	Italy
ON649708.1	41	66	2022	Portugal
ON649709.1	41	66	2022	Portugal
ON649710.1	41	65	2022	Portugal
ON649711.1	41	65	2022	Portugal
ON649712.1	41	66	2022	Portugal
ON649713.1	44	69	2022	Portugal
ON649714.1	41	65	2022	Portugal
ON649715.1	41	65	2022	Portugal
ON649716.1	42	66	2022	Portugal
ON649717.1	42	67	2022	Portugal
ON649718.1	41	66	2022	Portugal

ON649719.1	41	66	2022	Portugal
ON649720.1	41	66	2022	Portugal
ON649721.1	41	66	2022	Portugal
ON649722.1	41	66	2022	Portugal
ON649723.1	41	66	2022	Portugal
ON649724.1	41	66	2022	Portugal
ON649725.1	41	66	2022	Portugal
ON649879.1	41	66	2022	Israel
ON674051.1	27	37	2022	USA
ON675438.1	31	42	2022	USA
ON676703.1	41	66	2022	USA
ON676704.1	43	68	2022	USA
ON676705.1	42	67	2022	USA
ON676706.1	41	66	2022	USA
ON676707.1	31	41	2022	USA
ON676708.1	34	60	2022	USA
ON682263.4	41	65	2022	Germany
ON682264.4	41	65	2022	Germany
ON682265.4	41	66	2022	Germany
ON682266.2	43	68	2022	Germany
ON682267.2	41	66	2022	Germany
ON682268.3	43	68	2022	Germany
ON682269.3	41	65	2022	Germany
ON682270.2	41	66	2022	Germany
ON694329.1	41	66	2022	Germany
ON694330.1	45	70	2022	Germany
ON694331.1	42	67	2022	Germany
ON694332.1	41	66	2022	Germany
ON694333.1	41	66	2022	Germany
ON694334.1	42	67	2022	Germany
ON694335.1	43	68	2022	Germany
ON694336.1	41	66	2022	Germany
ON694337.1	42	67	2022	Germany
ON694338.1	41	66	2022	Germany
ON694339.1	42	67	2022	Germany
ON694340.1	41	66	2022	Germany
ON694341.2	42	67	2022	Germany
ON694342.1	43	68	2022	Germany
ON720848.1	43	66	2022	Spain
ON720849.1	45	69	2022	Spain
ON736420.2	41	66	2022	Canada

ON745215.1	41	66	2022	Italy
ON745225.1	42	67	2022	Spain
ON751962.1	44	69	2022	Portugal
ON754984.1	44	72	2022	Slovenia
ON754985.1	44	69	2022	Slovenia
ON754986.1	44	69	2022	Slovenia
ON754987.1	45	70	2022	Slovenia
ON754989.2	46	71	2022	Canada
ON755039.1	42	67	2022	France
ON755040.1	41	66	2022	France
ON755231.1	43	68	2022	Germany
ON755232.1	42	67	2022	Germany
ON755233.1	42	67	2022	Germany
ON755234.1	42	67	2022	Germany
ON755235.1	42	67	2022	Germany
ON755236.1	41	66	2022	Germany
ON755237.1	41	66	2022	Germany
ON755238.2	41	66	2022	Germany
ON755239.2	44	69	2022	Germany
ON755240.1	43	68	2022	Germany
ON755241.1	42	67	2022	Germany
ON755242.1	41	66	2022	Germany
ON755243.1	43	68	2022	Germany
ON755244.1	42	67	2022	Germany
ON755245.1	41	66	2022	Germany
ON755246.1	41	66	2022	Germany
ON755247.1	43	68	2022	Germany
ON755248.1	44	69	2022	Germany
ON755249.2	43	68	2022	Germany
ON755250.1	41	66	2022	Germany
ON755251.2	45	70	2022	Germany
ON755252.1	42	67	2022	Germany
ON755253.1	41	66	2022	Germany
ON755254.1	43	68	2022	Germany
ON755255.2	43	68	2022	Germany
ON755256.1	44	69	2022	Germany
ON780016.1	41	67	2022	Italy
ON780017.1	42	68	2022	Italy
ON782021.1	44	69	2022	Finland
ON782022.1	41	66	2022	Finland
ON782054.1	34	58	2022	Spain

ON782055.1	44	69	2022	Spain
ON792320.1	45	69	2022	Switzerland
ON792321.1	41	65	2022	Switzerland
ON792322.1	42	67	2022	Switzerland
ON803413.1	46	71	2022	Canada
ON803414.1	46	71	2022	Canada
ON803415.1	46	70	2022	Canada
ON803416.1	41	65	2022	Canada
ON803417.1	42	67	2022	Canada
ON803418.1	42	67	2022	Canada
ON803419.1	41	66	2022	Canada
ON803420.1	46	71	2022	Canada
ON803421.1	41	66	2022	Canada
ON803422.1	43	68	2022	Canada
ON803423.1	43	68	2022	Canada
ON803424.1	41	66	2022	Canada
ON803425.1	41	66	2022	Canada
ON803426.1	41	66	2022	Canada
ON803427.1	41	66	2022	Canada
ON803428.1	41	65	2022	Canada
ON803429.1	41	66	2022	Canada
ON803430.1	41	66	2022	Canada
ON803431.1	42	67	2022	Canada
ON803432.1	42	67	2022	Canada
ON803433.1	41	65	2022	Canada
ON803434.1	41	65	2022	Canada
ON803435.1	43	68	2022	Canada
ON803436.1	41	66	2022	Canada
ON803437.1	41	66	2022	Canada
ON803438.1	41	66	2022	Canada
ON803439.1	46	71	2022	Canada
ON803440.1	41	66	2022	Canada
ON803441.1	41	66	2022	Canada
ON803442.1	41	66	2022	Canada
ON803443.1	41	65	2022	Canada
ON803444.1	42	67	2022	Canada
ON808413.1	42	67	2022	UK
ON808414.1	41	66	2022	UK
ON808415.1	41	66	2022	UK
ON808416.1	42	67	2022	UK
ON808417.1	41	66	2022	UK

ON813251.2	42	66	2022	Germany
ON813252.2	41	66	2022	Germany
ON813253.2	42	67	2022	Germany
ON813254.2	43	68	2022	Germany
ON813255.2	41	66	2022	Germany
ON813256.2	42	67	2022	Germany
ON813257.2	44	69	2022	Germany
ON813258.2	42	67	2022	Germany
ON813259.2	43	68	2022	Germany
ON813260.2	44	69	2022	Germany
ON813261.2	44	69	2022	Germany
ON813262.2	42	67	2022	Germany
ON813263.2	43	68	2022	Germany
ON813264.2	42	67	2022	Germany
ON813265.2	41	66	2022	Germany
ON813266.2	42	67	2022	Germany
ON813267.2	41	66	2022	Germany
ON838178.1	44	69	2022	Portugal
ON838939.1	43	68	2022	Spain
ON838940.1	41	66	2022	Spain
ON843163.1	41	64	2022	Portugal
ON843164.1	41	67	2022	Portugal
ON843165.1	42	67	2022	Portugal
ON843166.1	43	68	2022	Portugal
ON843167.1	41	66	2022	Portugal
ON843168.1	44	69	2022	Portugal
ON843169.1	41	66	2022	Portugal
ON843170.1	42	67	2022	Portugal
ON843171.1	41	64	2022	Portugal
ON843172.1	41	66	2022	Portugal
ON843173.1	42	67	2022	Portugal
ON843174.1	42	67	2022	Portugal
ON843175.1	42	66	2022	Portugal
ON843176.1	41	66	2022	Portugal
ON843177.1	41	66	2022	Portugal
ON843178.1	41	66	2022	Portugal
ON843179.1	42	65	2022	Portugal
ON843180.1	41	64	2022	Portugal
ON843181.1	45	69	2022	Portugal
ON843182.1	42	66	2022	Portugal
ON853649.1	45	70	2022	Germany

ON853650.1	43	68	2022	Germany
ON853651.1	43	68	2022	Germany
ON853652.1	42	67	2022	Germany
ON853653.1	41	66	2022	Germany
ON853654.1	41	66	2022	Germany
ON853655.1	46	71	2022	Germany
ON853656.1	44	69	2022	Germany
ON853657.1	41	66	2022	Germany
ON853658.1	42	67	2022	Germany
ON853659.1	44	69	2022	Germany
ON853660.1	44	69	2022	Germany
ON853661.1	45	70	2022	Germany
ON853662.1	44	69	2022	Germany
ON853663.1	42	67	2022	Germany
ON853664.1	43	68	2022	Germany
ON853665.1	44	69	2022	Germany
ON853666.1	42	67	2022	Germany
ON853667.1	43	68	2022	Germany
ON853668.1	45	70	2022	Germany
ON853669.1	44	69	2022	Germany
ON853670.1	44	69	2022	Germany
ON853671.1	42	67	2022	Germany
ON853672.1	44	69	2022	Germany
ON853673.1	41	66	2022	Germany
ON853674.1	41	66	2022	Germany
ON853675.1	41	66	2022	Germany
ON853676.1	44	69	2022	Germany
ON853677.1	42	67	2022	Germany
ON853678.1	42	67	2022	Germany
ON853679.1	41	66	2022	Germany
ON853680.1	42	67	2022	Germany
ON853681.1	42	67	2022	Germany
ON853682.1	42	67	2022	Germany
ON872184.1	42	67	2022	Ireland
ON880413.1	45	70	2022	Brazil
ON880419.1	41	66	2022	Belgium
ON880420.1	41	66	2022	Belgium
ON880421.1	41	66	2022	Belgium
ON880422.1	41	66	2022	Belgium
ON880505.1	46	70	2022	Canada
ON880506.1	48	72	2022	Canada

ON880507.1	46	69	2022	Canada
ON880508.1	41	66	2022	Canada
ON880509.1	41	65	2022	Canada
ON880510.1	46	70	2022	Canada
ON880511.1	45	70	2022	Canada
ON880512.1	41	66	2022	Canada
ON880513.1	46	71	2022	Canada
ON880514.1	46	70	2022	Canada
ON880515.1	46	70	2022	Canada
ON880516.1	41	66	2022	Canada
ON880517.1	42	67	2022	Canada
ON880518.1	42	65	2022	Canada
ON880519.1	41	62	2022	Canada
ON880520.1	42	66	2022	Canada
ON880521.1	41	66	2022	Canada
ON880522.1	41	66	2022	Canada
ON880523.1	46	71	2022	Canada
ON880524.1	38	61	2022	Canada
ON880525.1	41	64	2022	Canada
ON880526.1	41	66	2022	Canada
ON880527.1	41	66	2022	Canada
ON880528.1	44	68	2022	Canada
ON880529.1	42	65	2022	Canada
ON880530.1	41	66	2022	Canada
ON880531.1	41	65	2022	Canada
ON880532.1	41	65	2022	Canada
ON880533.1	44	67	2022	Canada
ON880534.1	44	69	2022	Canada
ON880535.1	41	65	2022	Canada
ON880536.1	42	67	2022	Canada
ON880537.1	42	67	2022	Canada
ON880538.1	44	68	2022	Canada
ON880539.1	41	66	2022	Canada
ON880540.1	44	69	2022	Canada
ON880541.1	39	64	2022	Canada
ON880542.1	41	66	2022	Canada
ON880543.1	44	69	2022	Canada
ON880544.1	43	67	2022	Canada
ON880545.1	42	67	2022	Canada
ON880546.1	41	66	2022	Canada
ON880547.1	45	70	2022	Canada

ON880548.1	43	68	2022	Canada
ON880549.1	44	69	2022	Canada

Table S5. Genomes used in this analysis, with APOBEC3 counts relative to MT903345 (only genomes from 2019 onwards) and KJ64261 (all genomes).

```

################################
# © Crown copyright Dstl 2021 #
# OFFICAL                    #
################################

### Script designed to take output fasta files and BEDTools generated coverage data and replace bases with 'N' characters if coverage is below a specified value
## Command use: consensus_coverage.R coverage.csv consensus.fasta coverage_depth
# coverage.csv: generated from BEDTools (all positions)
# consensus.fasta: sequence must be in a single-line format
# coverage_depth: Coverage depth cut-off. Depths below this will be replaced with a 'N'


## Allowing arguments to be loaded from the command line
args <- commandArgs(TRUE)

## Loading data
coverage <- read.csv(args[1],sep="\t",header=FALSE)
# Loading consensus
consensus <- read.table(args[2])
# Loading user defined coverage depth
coverage_depth=as.numeric(args[3])
# output location
output=args[4]
# name to use
name=args[5]

print(paste0("Coverage cut of is: ", coverage_depth))

##### Starting analysis #####
# Removing fasta name
consensus_original <- as.character(consensus[2,])
# Calculating length of consensus
consensus_length=nchar(consensus_original)
# Splitting strings so each base is a separate vector
consensus <- strsplit(consensus_original,"")

# Calculating nrows in coverage
coverage_length=nrow(coverage)

```

if (as.numeric(consensus_length) == as.numeric(coverage_length)) {
# Adding consensus sequence to the coverage data frame
coverage[,4] <- consensus
# Adding colnames
colnames(coverage) <- c("Refernece","Position","Coverage","Sequence")
# Changing bases to N if coverage is below x
coverage[,4] <- ifelse(coverage$Coverage < coverage_depth, "N", coverage$Sequence)
# Collapsing consensus back into a single string
consensus <- as.character(paste(coverage$Sequence,collapse=''))
# Adding in the consensus name
consensus <- rbind(paste0(">",name,"_medaka"),consensus)
# Generating consensus sequence
write.table(consensus,paste0(output,"/",name,"_consensus.fasta"),row.names=FALSE,col.names=FALSE,quote=FALSE)
# Writing modified BEDTools table
write.csv(coverage,paste0(output,"/",name,"_coverage.csv"),row.names=FALSE)
} else {
print ("")
print("Consensus length does not match the coverage depth data optained from the alignment")
print("This is most likley due to a INDEL being introduced from the latest Medaka polish")
print("Further medaka polishing rounds may resolve this")
print("")
write.table(consensus_original,paste0(output,"/",name,"_NoCoverageFilter_consensus.fasta"),row.names=FALSE,col.names=FALSE,quote=FALSE)
}

Table S6. In-house script for consensus_coverage.R – Loads in a coverage.csv file (argument 1) (i.e. coverage at each position, generated from Bedtools) & consensus sequence (argument 2). If the coverage is less than inputted coverage depth (argument 3), then the base is replaced with a ‘N’. The sequences within the fasta file must be single line. It can be used via the following command line:
consensus_coverage.R: *coverage.csv consensus.fasta coverage_depth*

```
## Script info

#################################
# © Crown copyright Dstl 2021 #
# OFFICAL                 #
#################################

## Designed to investigate APOBEC3 style mutations within input monkeypox genomes aligned to a reference sequence
# The script uses a sliding window appraoch (window size = 2, step size = 1) to identify 'tc > tt', 'ga > aa', 'cc > ct' & 'gg > ag' mutations

# Command use: Rscript APOBEC3_dinucleotide.R aligned.fasta refName altName
# fasta data input must be aligned (single line) and the same size

# Script version 1.1
# Last modified: 06/07/2022

## Loading data
# Passed from command line
args <- commandArgs(TRUE)

# loading the sequence
fasta <- read.table(args[1], sep="\t") # loading aligned fasta
refName <- args[2]
altName <- args[3]
genomeName <- gsub(">","",fasta[seq(1,nrow(fasta),2),]) # using fasta headers as names

## Splitting bases into a dataframe
ref <- as.data.frame(strsplit(tolower(fasta[2,]),"")) # extracting ref seq and converting to lower case
bases <- as.data.frame(matrix(ncol=nrow(fasta),nrow=nrow(ref))) # creating a data frame to hold the data. Empty columns will be present but these will be removed later
bases[,1] <- ref # adding reference to data frame

for (i in seq(4,nrow(fasta),2)) { # Looping through aligned data and extracting
  bases[,i] <- as.data.frame(strsplit(tolower(fasta[i,]),""))
}
empty_columns <- sapply(bases,function(x) all(is.na(x) | x == "")) # finding empty columns
bases <- bases[, !empty_columns] # removing empty columns
colnames(bases) <- c(refName,altName)

window <- 2 # to use if the window and step sizes need to change
```

```
step <- 1

# Creating data frames for APOBEC3 & APOBEC3G mutations
APOBEC3_mutations <- as.data.frame(matrix(ncol=2,nrow=0))
APOBEC3_mutations_rev <- as.data.frame(matrix(ncol=2,nrow=0))
APOBEC3_mutations_main <- as.data.frame(matrix(ncol=2,nrow=0)) # contains both rev and comp

APOBEC3G_mutations <- as.data.frame(matrix(ncol=2,nrow=0))
APOBEC3G_mutations_rev <- as.data.frame(matrix(ncol=2,nrow=0))
APOBEC3G_mutations_main <- as.data.frame(matrix(ncol=2,nrow=0)) # contains both rev and comp

colnames(APOBEC3_mutations) <- c("position",colnames(bases[2]))
colnames(APOBEC3_mutations_rev) <- c("position",colnames(bases[2]))
colnames(APOBEC3_mutations_main) <- c("position",colnames(bases[2]))

colnames(APOBEC3G_mutations) <- c("position",colnames(bases[2]))
colnames(APOBEC3G_mutations_rev) <- c("position",colnames(bases[2]))
colnames(APOBEC3G_mutations_main) <- c("position",colnames(bases[2]))

print("")
print(paste0("Analysing sample ",colnames(bases[2])))

## Looping through the first alt sequence and extracting data
for (i in seq(1,nrow(bases),step)) { # looping through each base

  window_select <- bases[c(i,i+c(window-1)),] # selecting the current base and the base after it
  ref_window_1 <- window_select[1,1]
  ref_window_2 <- window_select[2,1]
  ref_window <- paste(ref_window_1,ref_window_2,sep="") # combining into a string

  alt_window_1 <- window_select[1,2]
  alt_window_2 <- window_select[2,2]
  alt_window <- paste(alt_window_1,alt_window_2,sep="") # combining into a string

  ## Looking for APOBEC3 mutations
  if ( (ref_window == "tc")&(alt_window == "tt") ){
    print(paste0("APOBEC3 style mutation found at position ",i))
    APOBEC3_mutations[nrow(APOBEC3_mutations)+1,] <- c(i,alt_window)
    APOBEC3_mutations_main[nrow(APOBEC3_mutations_main)+1,] <- c(i,alt_window) # adding to main data frame
  }
  ## Looking for APOBEC3 reverse mutations
```

if ((ref_window == "ga")&(alt_window == "aa")){
print(paste0("APOBEC3 style mutation (reverse) found at position ",i))
APOBEC3_mutations_rev[nrow(APOBEC3_mutations_rev)+1,] <- c(i,alt_window)
APOBEC3_mutations_main[nrow(APOBEC3_mutations_main)+1,] <- c(i,alt_window) # adding to main data frame
}
Looking for APOBEC3G mutations
if ((ref_window == "cc")&(alt_window == "ct")){
print(paste0("APOBEC3G style mutation found at position ",i))
APOBEC3G_mutations[nrow(APOBEC3G_mutations)+1,] <- c(i,alt_window)
APOBEC3G_mutations_main[nrow(APOBEC3G_mutations_main)+1,] <- c(i,alt_window) # adding to main data frame
}
Looking for APOBEC3G reverse mutations
if ((ref_window == "gg")&(alt_window == "ag")){
print(paste0("APOBEC3G style mutation (reverse) found at position ",i))
APOBEC3G_mutations_rev[nrow(APOBEC3G_mutations_rev)+1,] <- c(i,alt_window)
APOBEC3G_mutations_main[nrow(APOBEC3G_mutations_main)+1,] <- c(i,alt_window) # adding to main data frame
}
}
Writing data to csv
write.csv(APOBEC3_mutations_main,"APOBEC3_mutations.csv",row.names = FALSE)
write.csv(APOBEC3G_mutations_main,"APOBEC3G_mutations.csv",row.names = FALSE)

Table S7. In-house script for APOBEC3_dinucleotide.R – Uses a sliding window approach (window size=2, step size = 1) to identify the number of APOBEC3 differences between two aligned sequences (a reference sequence, e.g. Nigeria 1971 and any other mpox sequence). The sequences within the fasta file must be single line (argument 1). Also requires the reference name (argument 2) & alternative name (argument 3). It can be used via the following command line:
APOBEC3_dinucleotide.R *aligned.fasta consensus_name alt_name*
