# Supplementary material for: Distinct monkeypox virus lineages co-circulating in humans before 2022
Source: Nat Med. 2023 Sep 14;29(9):2317–24. doi: 10.1038/s41591-023-02456-8 (PMC10504077; doi:10.1038/s41591-023-02456-8)
Supplement: Supplementary file 2 — Reporting Summary [file 41591_2023_2456_MOESM2_ESM.pdf]

nature portfolio

Corresponding author(s): *David Ulaeto and Ifedayo Adetifa*

Last updated by author(s): *May 12, 2023*

Reporting Summary

Nature Portfolio wishes to improve the reproducibility of the work that we publish. This form provides structure for consistency and transparency in reporting. For further information on Nature Portfolio policies, see our Editorial Policies and the Editorial Policy Checklist.

Statistics

For all statistical analyses, confirm that the following items are present in the figure legend, table legend, main text, or Methods section.

n/a	Confirmed	
☐	☒	The exact sample size (*n*) for each experimental group/condition, given as a discrete number and unit of measurement
☐	☒	A statement on whether measurements were taken from distinct samples or whether the same sample was measured repeatedly
☐	☒	The statistical test(s) used AND whether they are one- or two-sided<br>*Only common tests should be described solely by name; describe more complex techniques in the Methods section.*
☐	☒	A description of all covariates tested
☐	☒	A description of any assumptions or corrections, such as tests of normality and adjustment for multiple comparisons
☐	☒	A full description of the statistical parameters including central tendency (e.g. means) or other basic estimates (e.g. regression coefficient) AND variation (e.g. standard deviation) or associated estimates of uncertainty (e.g. confidence intervals)
☐	☒	For null hypothesis testing, the test statistic (e.g. *F*, *t*, *r*) with confidence intervals, effect sizes, degrees of freedom and *P* value noted<br>*Give P values as exact values whenever suitable.*
☒	☐	For Bayesian analysis, information on the choice of priors and Markov chain Monte Carlo settings
☒	☐	For hierarchical and complex designs, identification of the appropriate level for tests and full reporting of outcomes
☒	☐	Estimates of effect sizes (e.g. Cohen's *d*, Pearson's *r*), indicating how they were calculated

Our web collection on statistics for biologists contains articles on many of the points above.

Software and code

Policy information about availability of computer code

Data collection	*No software was used for data collection*
Data analysis	guppy (version 3.6) (ONT); CANU (version 2.1.1); BLASTn (version 2.12.0); Medaka (version 1.2.2) (https://github.com/nanoporetech/medaka); BEDtools (version 2.27.1); MAFFT (version 7.453); IQ-Tree (version 2.1.3). References for software and algorithms are provided in the manuscript.

For manuscripts utilizing custom algorithms or software that are central to the research but not yet described in published literature, software must be made available to editors and reviewers. We strongly encourage code deposition in a community repository (e.g. GitHub). See the Nature Portfolio guidelines for submitting code & software for further information.

Data

Policy information about availability of data

All manuscripts must include a data availability statement. This statement should provide the following information, where applicable:

- Accession codes, unique identifiers, or web links for publicly available datasets
- A description of any restrictions on data availability
- For clinical datasets or third party data, please ensure that the statement adheres to our policy

Genomes for the 18 isolates described here are deposited in GenBank; accession numbers are listed in Table S5.

Human research participants

Policy information about studies involving human research participants and Sex and Gender in Research.

Reporting on sex and gender	patient gender is reported in table 1
Population characteristics	n/a patients were not recruited. Samples were from archived materials, and all available samples were used regardless of demographic
Recruitment	n/a patients were not recruited. Samples were from archived materials, and all available samples were used. Ethical approval for the study was obtained subsequent to sample collection
Ethics oversight	National Health Research Ethics Committee of Nigeria (NHREC) (Ref: NHREC/01/01/2007-19 /03/2019)

Note that full information on the approval of the study protocol must also be provided in the manuscript.

Field-specific reporting

Please select the one below that is the best fit for your research. If you are not sure, read the appropriate sections before making your selection.

☒ Life sciences ☐ Behavioural & social sciences ☐ Ecological, evolutionary & environmental sciences

For a reference copy of the document with all sections, see nature.com/documents/nr-reporting-summary-flat.pdf

Life sciences study design

All studies must disclose on these points even when the disclosure is negative.

Sample size	18 genomes are reported because 18 was the number available. For other post 2017 sequences, all available sequences were included up to a cut-off date of June 18th 2022
Data exclusions	No data were excluded. For 2022 sequences statistical analyses were performed with with all available 2022 sequences, and separately excluding 2 sequences from a separate lineage. This is described in the manuscript.
Replication	n/a replication is not appropriate because replicate samples / additional sequencing of samples was not available
Randomization	n/a, as all available samples were sequenced
Blinding	na, as all available samples were sequenced and none were subject to experimental procedures before extraction and sequencing

Reporting for specific materials, systems and methods

We require information from authors about some types of materials, experimental systems and methods used in many studies. Here, indicate whether each material, system or method listed is relevant to your study. If you are not sure if a list item applies to your research, read the appropriate section before selecting a response.

Materials & experimental systems

n/a	Involved in the study	
☒	☐	Antibodies
☒	☐	Eukaryotic cell lines
☒	☐	Palaeontology and archaeology
☒	☐	Animals and other organisms
☒	☐	Clinical data
☒	☐	Dual use research of concern

Methods

n/a	Involved in the study	
☒	☐	ChIP-seq
☒	☐	Flow cytometry
☒	☐	MRI-based neuroimaging
